# Supplementary material for: Calcium sensing by the STIM1 ER-luminal domain
Source: Nat Commun. 2018 Oct 31;9:4536. doi: 10.1038/s41467-018-06816-8 (PMC6208404; doi:10.1038/s41467-018-06816-8)
Supplement: Supplementary file 1 — Supplementary Information [file 41467_2018_6816_MOESM1_ESM.pdf]

SUPPLEMENTARY INFORMATION

Calcium sensing by the STIM1 ER-luminal domain

Gudlur et al

Supplementary Fig. 1

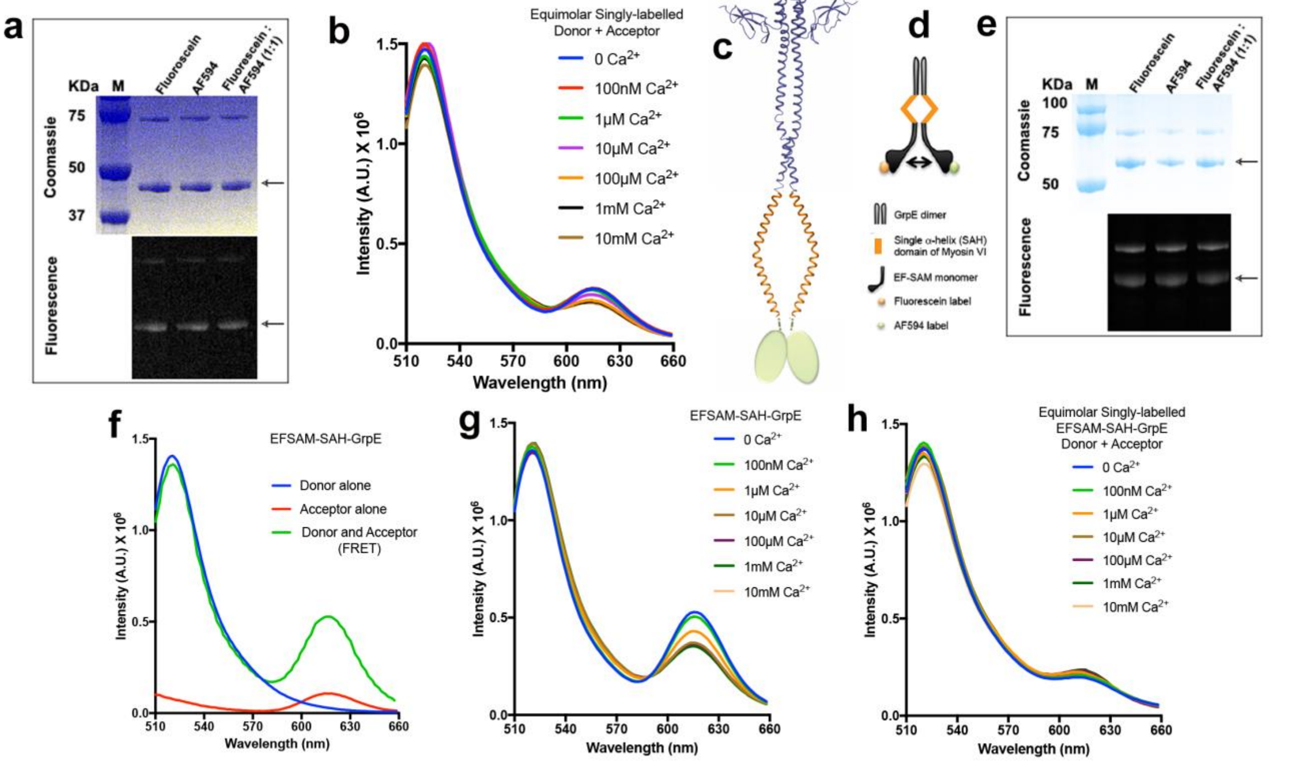

Supplementary Figure 1. EFSAM-GrpE and EFSAM-SAH-GrpE intradimer FRET measurements. a, SDS-polyacrylamide gel analysis of fluorescent dye-labeled EFSAM-GrpE proteins used for energy transfer experiments. Lanes with single- or double-labeled EFSAM-GrpE, stained with Coomassie Brilliant Blue in the top panel and the same lanes illuminated with UV light in the bottom panel, to assess labeling. Arrows mark the fully denatured monomer band. b, Fluorescence spectra (λ_{ex}= 420 nm) of an equimolar mix of separately labeled fluorescein- and AF594-labeled EFSAM-GrpE proteins incubated with varying amounts of Ca^{2+}. c, In EFSAM-SAH-GrpE, a single α-helix linker (orange) comparable in length to CC1 is inserted between the EFSAM domain and GrpE. d, Schematic of chemical labeling of EFSAM-SAH-GrpE, depicting the case where individual monomers are labeled with Fluorescein and AF594. Other possible combinations in the random labeling approach used here are not shown. e, SDS-polyacrylamide gel analysis of fluorescent dye-labeled EFSAM-SAH-GrpE proteins used for energy transfer experiments. Lanes with single- or double-labeled EFSAM-SAH-GrpE, stained with Coomassie Brilliant Blue in the top panel and the same lanes illuminated with UV light in the bottom panel, to assess labeling. Arrows mark the fully denatured monomer band. f, Fluorescence emission spectra (λ_{ex}= 420 nm) of EFSAM-SAH-GrpE labeled with fluorescein (Donor alone), AF594 (Acceptor alone) and double-labeled with both dyes. All measurements were made in Ca^{2+}-free buffer. g, Fluorescence emission spectra (λ_{ex}= 420 nm) of double-labeled EFSAM-SAH-GrpE with varying amounts of Ca^{2+}. h, Fluorescence emission spectra (λ_{ex}= 420 nm) of an equimolar mix of separately labeled fluorescein- and AF594-labeled EFSAM-SAH-GrpE proteins titrated with varying amounts of Ca^{2+}. Data in all panels representative of two experiments each.

Supplementary Fig.2

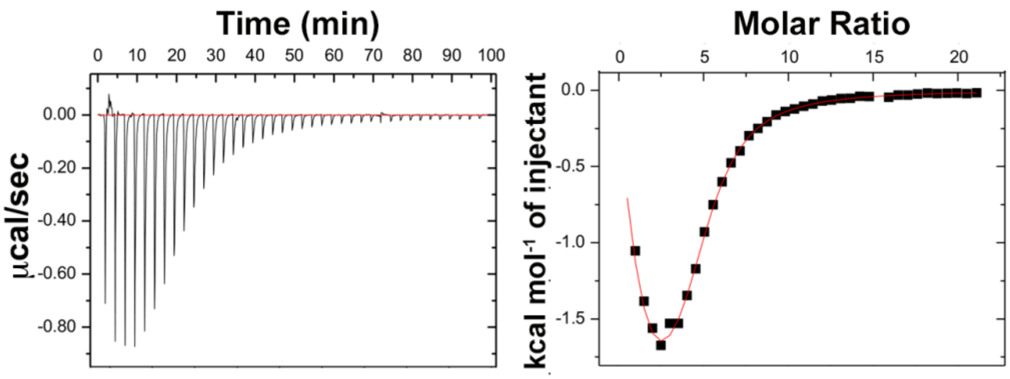

Supplementary Figure 2. ITC analysis of wildtype EFSAM-GrpE in the presence of 2 mM Mg^{2+}. Left panel, Heat changes measured after injecting 1 µl aliquots of 10 mM Ca^{2+} into a sample cell containing initially 100 µM protein. Right panel, Integrated binding isotherm as a function of molar ratio (Ca^{2+}:protein) after subtracting heats of dilution. The similarity to Figure 3a indicates that the sites detected are Ca^{2+}-specific.

Supplementary Fig.3

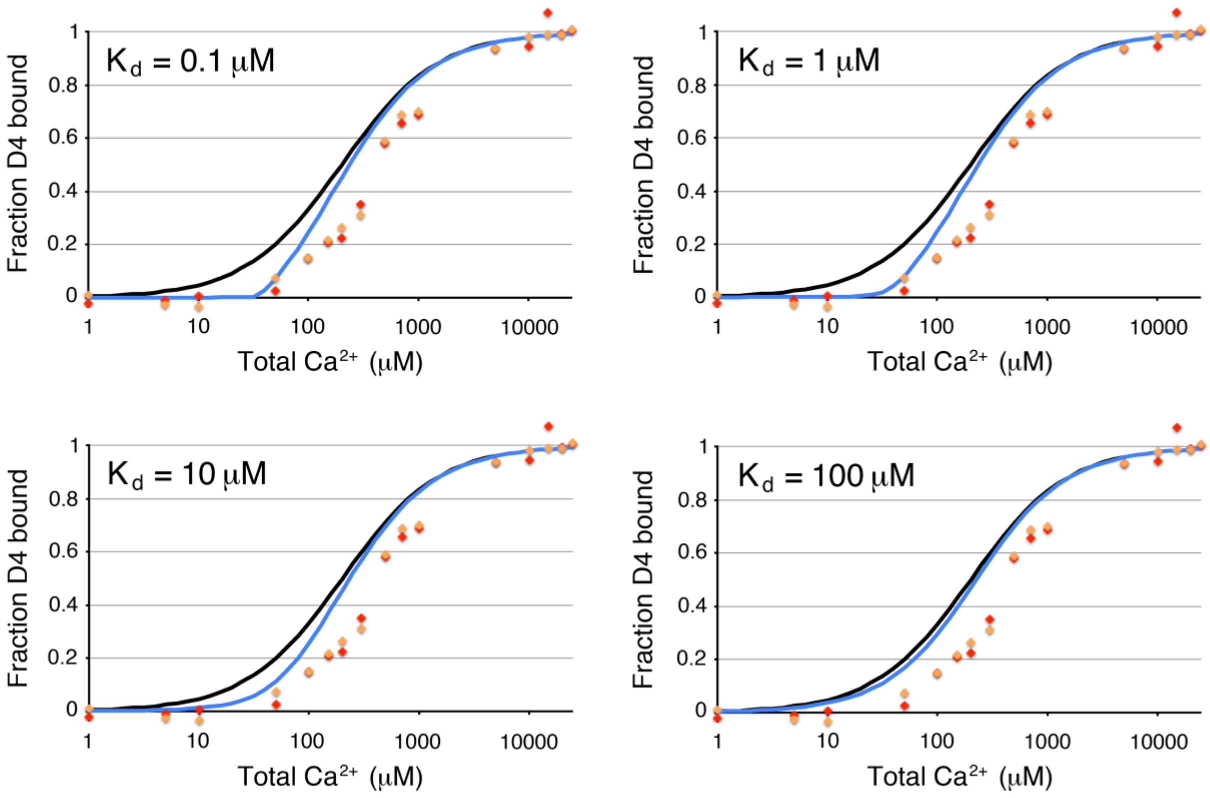

Supplementary Figure 3. Calculated D4 fluorescence competition curves. Calculated Ca^{2+}-binding curves for D4 sensor at trace levels in the presence of a competitor (35 μM) with a single Ca^{2+} binding site of K_d 0.1, 1, 10, or 100 μM (blue curves) are compared with the actual experimental data from two experiments (orange and red diamonds). The data are the same as in Fig. 3g, except that there the average of the two experiments is plotted. The fitted Ca^{2+} titration of D4 in the presence of GrpE (black curves) is shown for reference.

Supplementary Fig. 4

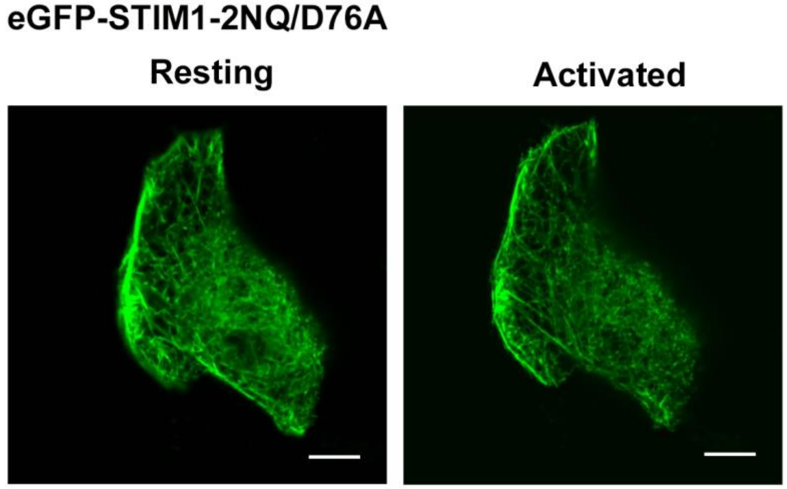

Supplementary Figure 4. STIM1(D76A)-2NQ fails to localize at puncta. Confocal micrographs of a HeLa cell expressing eGFP-STIM1(D76A)-2NQ, at rest (left panel) and after store depletion with 1 μM thapsigargin (TG) (right panel). Scale bar, 5 μm.

Supplementary Fig. 5

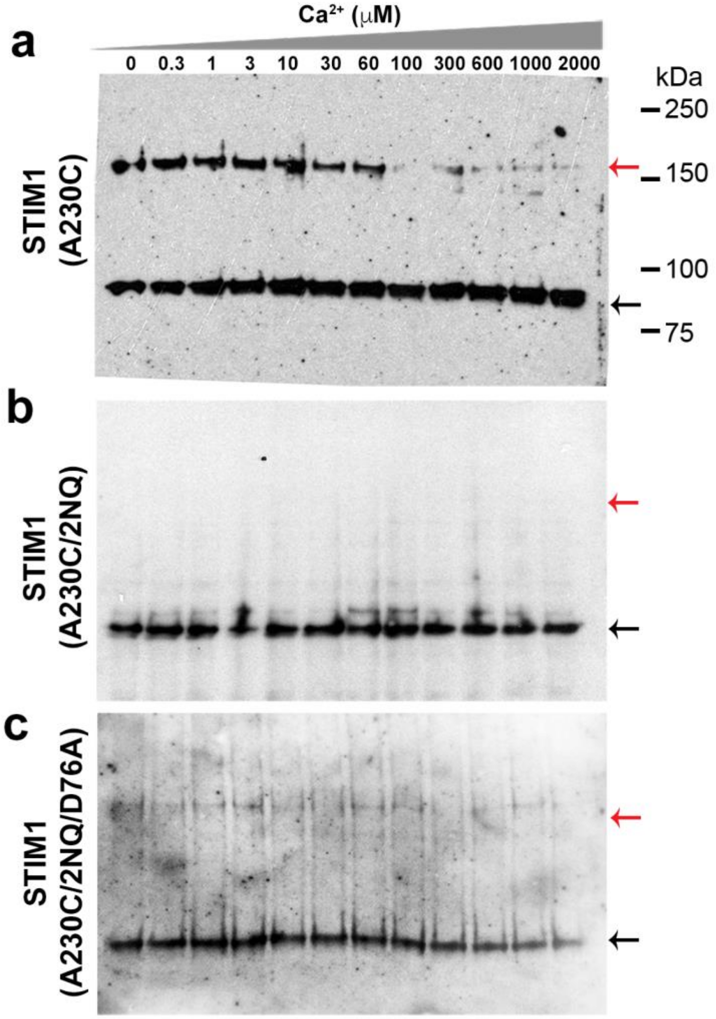

Supplementary Figure 5. STIM1-2NQ fails to respond to lowered Ca^{2+} concentration *in vitro*. Western blots showing crosslinking of STIM1(A230C) (a), STIM1(A230C)-2NQ (b), and STIM1(D76A/A230C)-2NQ (c) in cellular membranes incubated at the specified Ca^{2+} concentrations. Black arrows mark the STIM1 monomer bands, and red arrows mark the dimer bands. Panels (a) and (b) are representative of three experiments, and panel (c), of two experiments.

Supplementary Fig.6

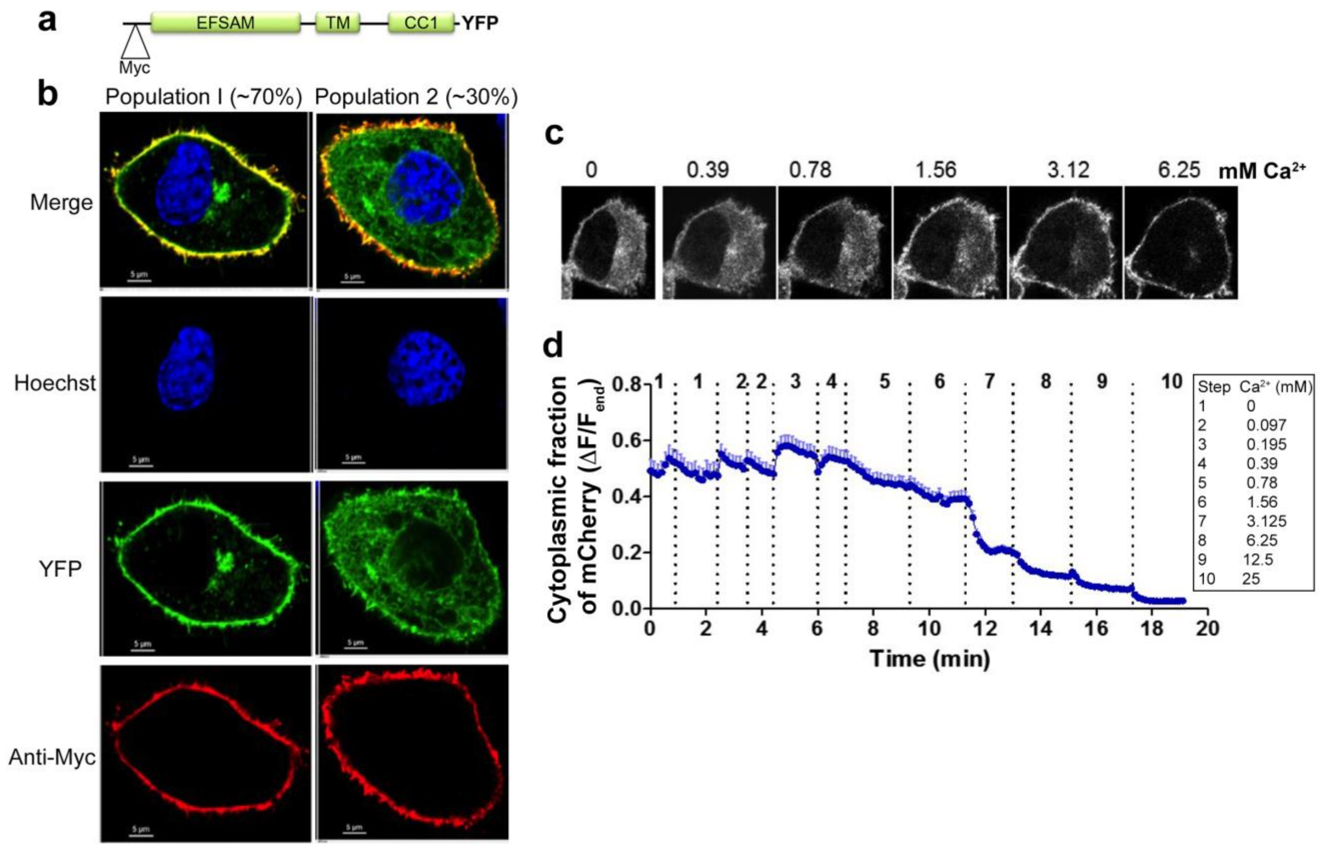

Supplementary Figure 6. CC1-SOAR binding assay in cells. a, STIM2 Myc-EFSAM-TM-CC1-YFP construct. b, Anti-Myc staining of living HeLa cells expressing STIM2 Myc-EFSAM-TM-CC1-YFP, illustrating the cellular localization of the STIM2 fragment and its orientation in the plasma membrane. Typically two populations of cells are observed, a major fraction in which STIM2 EFSAM-TM-CC1 is predominantly localized in the plasma membrane, and a minor fraction with both plasma membrane and ER localization. c, Confocal micrograph of a representative cell, showing the progressive recruitment of mCherry-CAD to CC1 at the plasma membrane in response to sequential stepwise increases in extracellular Ca^{2+} concentration. d, Ca^{2+} concentration dependence of mCherry-CAD recruitment to CC1, plotted as the decrease in cytoplasmic mCherry-CAD (n=17).

Supplementary Fig. 7

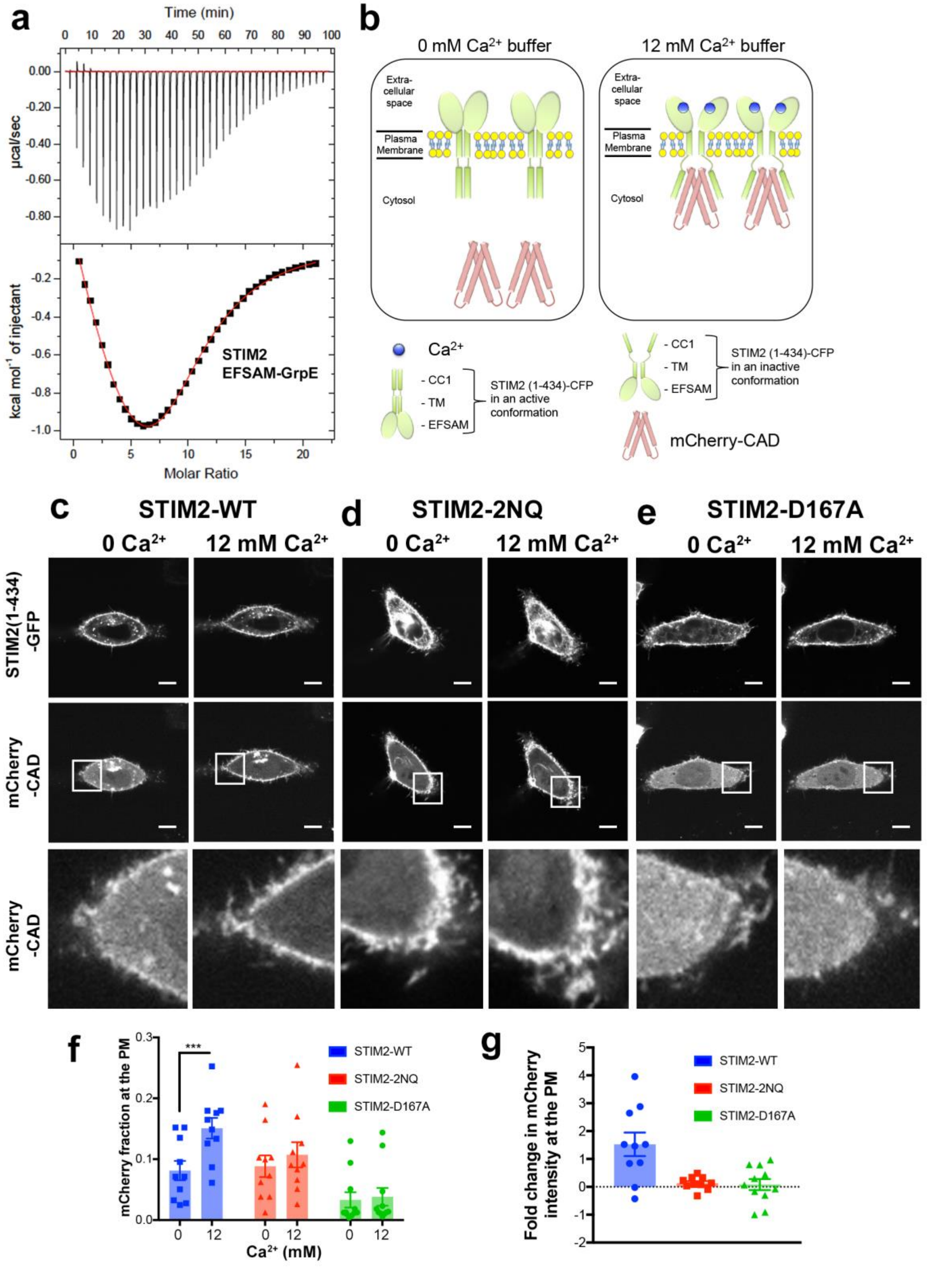

Supplementary Figure 7. STIM2-2NQ fails to release SOAR/CAD in cells. a, ITC analysis of Ca^{2+} binding to STIM2 EFSAM-GrpE, showing raw heat changes corresponding to 1 μl injections of 10 mM Ca^{2+} into a sample cell containing initially 100 μM protein in the top panel, and integrated heat change after subtracting heats of dilution at varying Ca^{2+}:protein molar ratios in the bottom panel. b, Schematic representation of the CC1-SOAR/CAD interaction assay. c–e, Confocal sections at the level of the nucleus of HeLa cells co-expressing WT STIM2(1-434)-GFP (c, upper panels) or the 2NQ variant (d, upper panels) or the D167A variant (e, upper panels), together with mCherry-CAD (middle panels, and at higher magnification in lower panels). Relocalization of mCherry-CAD was assessed after changing the extracellular solution from no Ca^{2+} (left panels of each set) to 12 mM Ca^{2+} (right panels of each set). Scale bars, 5 μm. f, Bar graph of the fraction of total mCherry-CAD intensity in a circumferential region near the plasma membrane (PM) under the conditions of panels (c)–(e). The fluorescence signal in the circumferential 'plasma membrane' region may include a contribution from unbound cytoplasmic mCherry-CAD. Data from STIM2-WT, n = 11 cells; STIM2-2NQ, n = 10 cells; STIM2-D167A, n = 10 cells. ***, $p < 0.05$ for STIM2-WT; the differences for STIM2-2NQ and STIM2-D167A were not statistically significant. g, Bar graph showing the fold change in mCherry-CAD intensity near the plasma membrane, relative to its initial value, after changing extracellular solution from no Ca^{2+} to 12 mM Ca^{2+}.

Supplementary Fig. 8

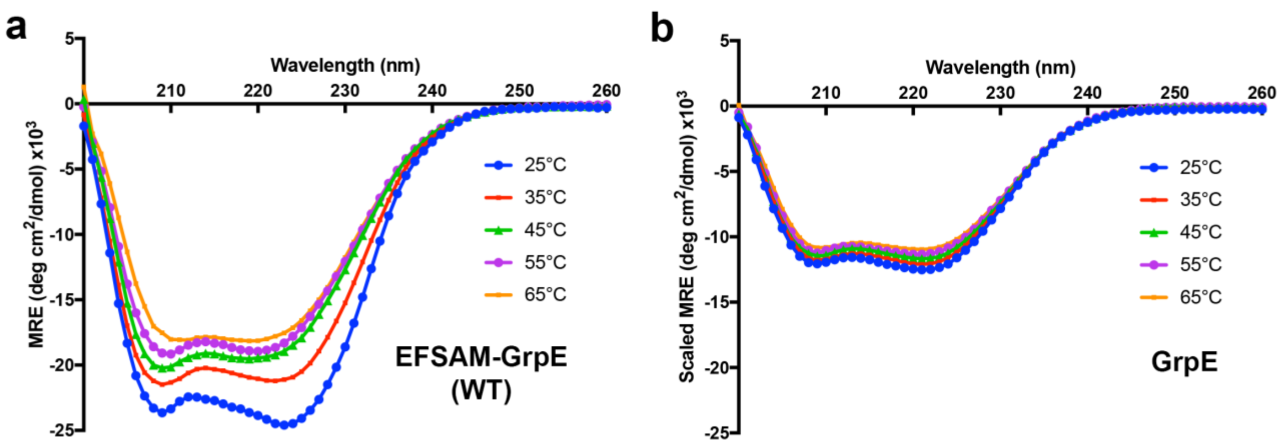

Supplementary Figure 8. Additional data related to the CD measurements. a, The far-UV CD spectrum of EFSAM-GrpE exhibited a decrease in the negative bands characteristic of α-helical secondary structure with increasing temperature in the range 25C to 65C. MRE is the mean residue ellipticity. b, The spectrum of GrpE over the same temperature range exhibits little change. The MRE values determined for GrpE have been multiplied by the scaling factor 174/370— which is (number of residues in GrpE) / (number of residues in EFSAM-GrpE)— to depict the expected contribution of GrpE to the CD spectrum of the full fusion protein. The concentration of protein in each sample was 5 μM.

Supplementary Fig.9

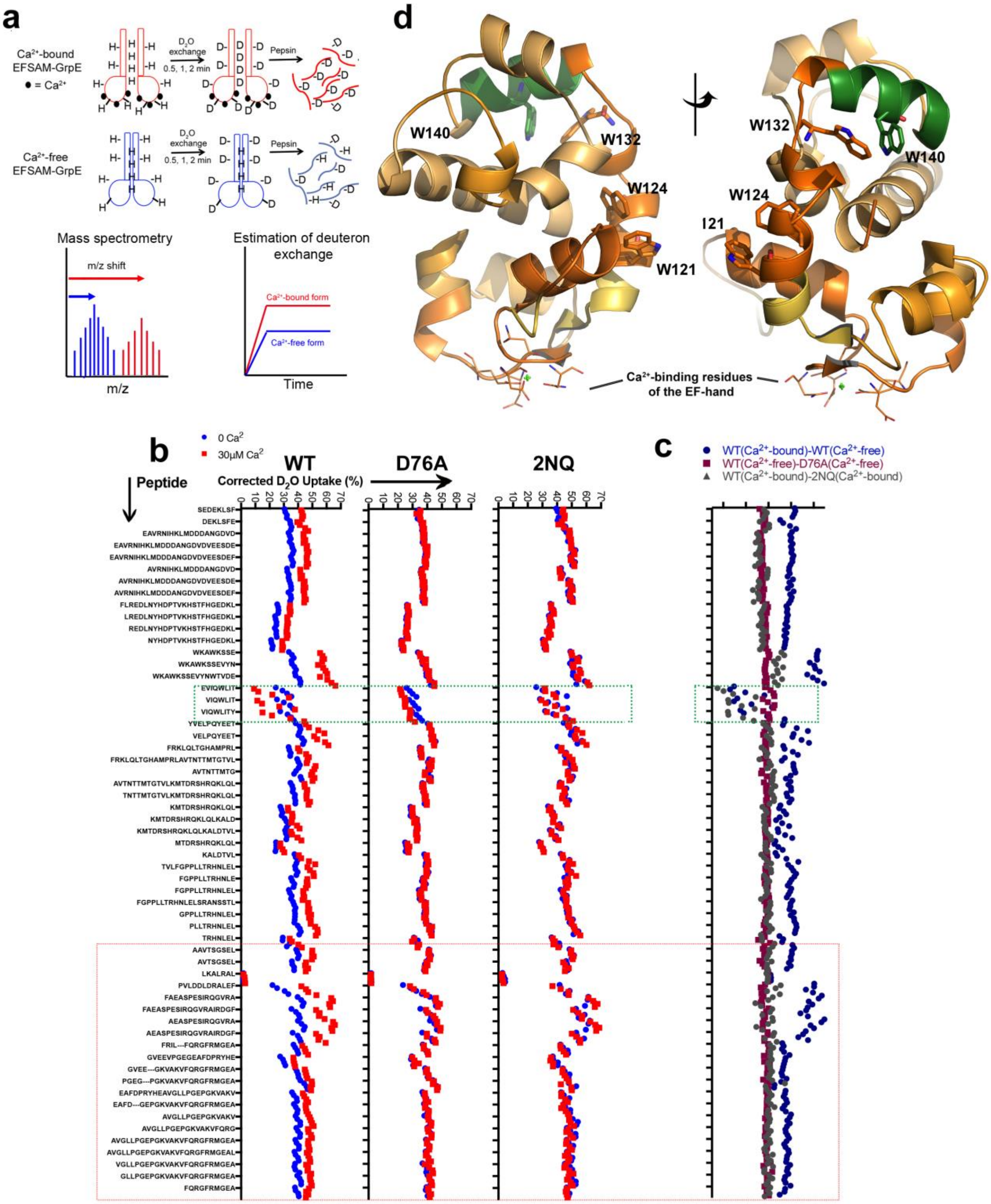

Supplementary Figure 9. EFSAM remains structured despite loss of Ca^{2+}. a, Graphic diagram of the HDX-MS work-flow used in this study. b, Deuterium exchange plots of the full repertoire of observed EFSAM-GrpE peptides, including GrpE peptides, in 0 Ca^{2+} and 30 μM Ca^{2+}, at 0.5, 1, 2 and 5 min time points. c, Differences in deuterium exchange for the same pairs of conditions examined in Fig. 5i with data plotted for the complete set of time points (0.5, 1, 2 and 5 min). Low-exchanging peptides in Ca^{2+}-bound condition from the EFSAM region around EVIQWLIT are boxed (green dotted box). Peptic peptides derived from GrpE are enclosed by a red dotted box. Note that the conformational change of wildtype EFSAM propagates to the distant β-sheet domain of GrpE. Deuterium exchange of GrpE fused to EFSAM(D76A) resembles that for Ca^{2+}-free wildtype EFSAM-GrpE, and exchange of GrpE fused to EFSAM(2NQ) resembles that for Ca^{2+}-bound wildtype EFSAM-GrpE, establishing that H-D exchange in the β-sheet domain of GrpE is sensitive to the conformation of EFSAM rather than to the absence or presence of Ca^{2+}. We have not explored the basis for the propagated conformational change, but it is likely that the EFSAM rearrangement exerts this effect through the contiguous α-helices of GrpE. It is known that the N-terminal α-helices of native GrpE are physically coupled to the β-sheet domain in *E coli* GrpE, and that thermal sensing in *T thermophilus* GrpE likewise involves a conformational change that increases protease sensitivity in both regions[1–3]. Data are the averages of triplicate measurements. d, EFSAM structure (PDB ID: 2K60) colored according to the change in deuterium exchange rates in the WT protein between the Ca^{2+}-free state and the Ca^{2+}-bound state. Two rotational views shown. Tryptophan residues are marked.

Supplementary Fig.10

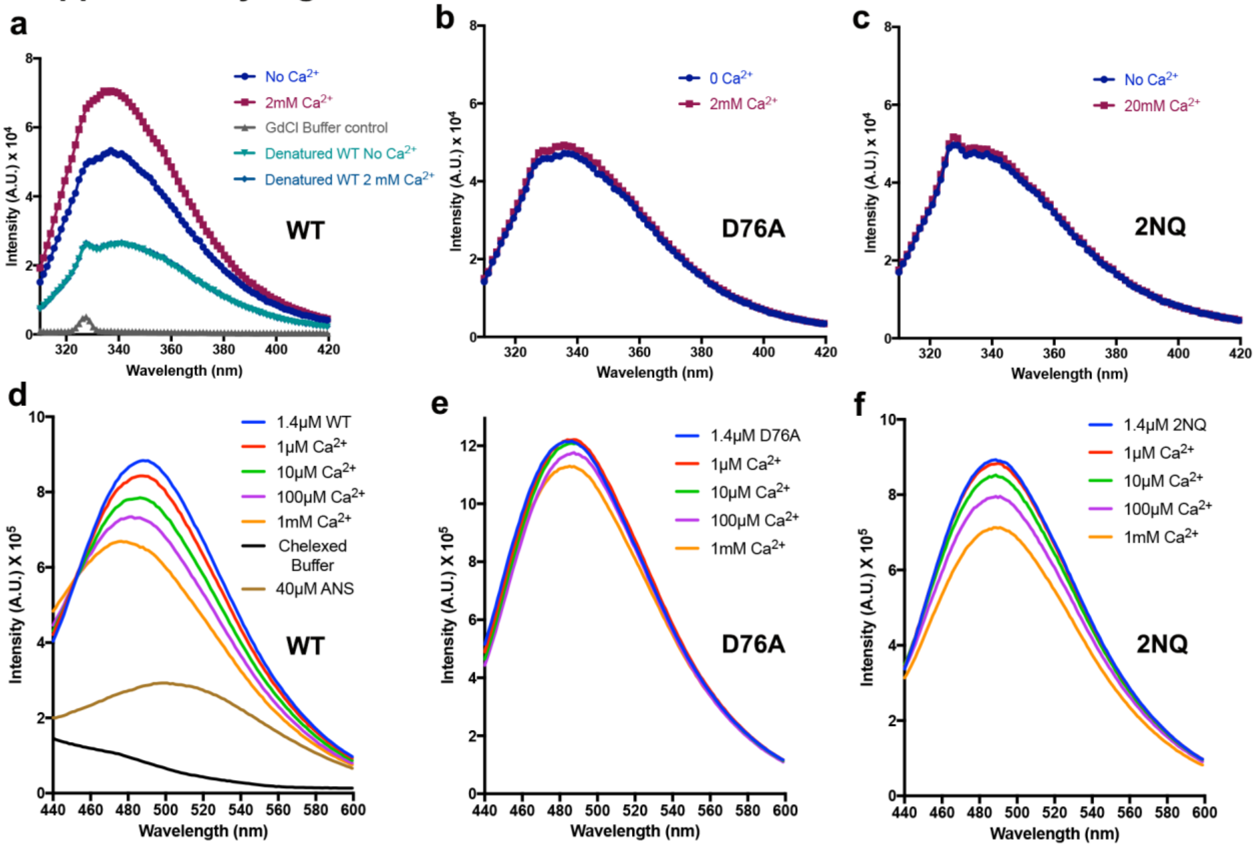

Supplementary Figure 10. EFSAM conformations probed by tryptophan and ANS fluorescence. a–c, Intrinsic tryptophan fluorescence spectra (λ_{ex}= 295 nm) of wildtype EFSAM-GrpE (WT; a), EFSAM(D76A)-GrpE (D76A; b), and EFSAM-2NQ-GrpE (2NQ; c). 6M guanidine chloride (GdCl) denaturation control spectra for EFSAM-GrpE-WT are shown in panel (a). d–f, Fluorescence spectra of ANS (λ_{ex}= 380 nm) in the presence of wildtype, D76A, and 2NQ EFSAM-GrpE proteins (1.4 μM) and the indicated concentrations of Ca^{2+}. ANS emission in the absence of any protein is shown as a brown curve in panel (d). Panels (d)–(f) are representative of two experiments.

Supplementary Fig. 11

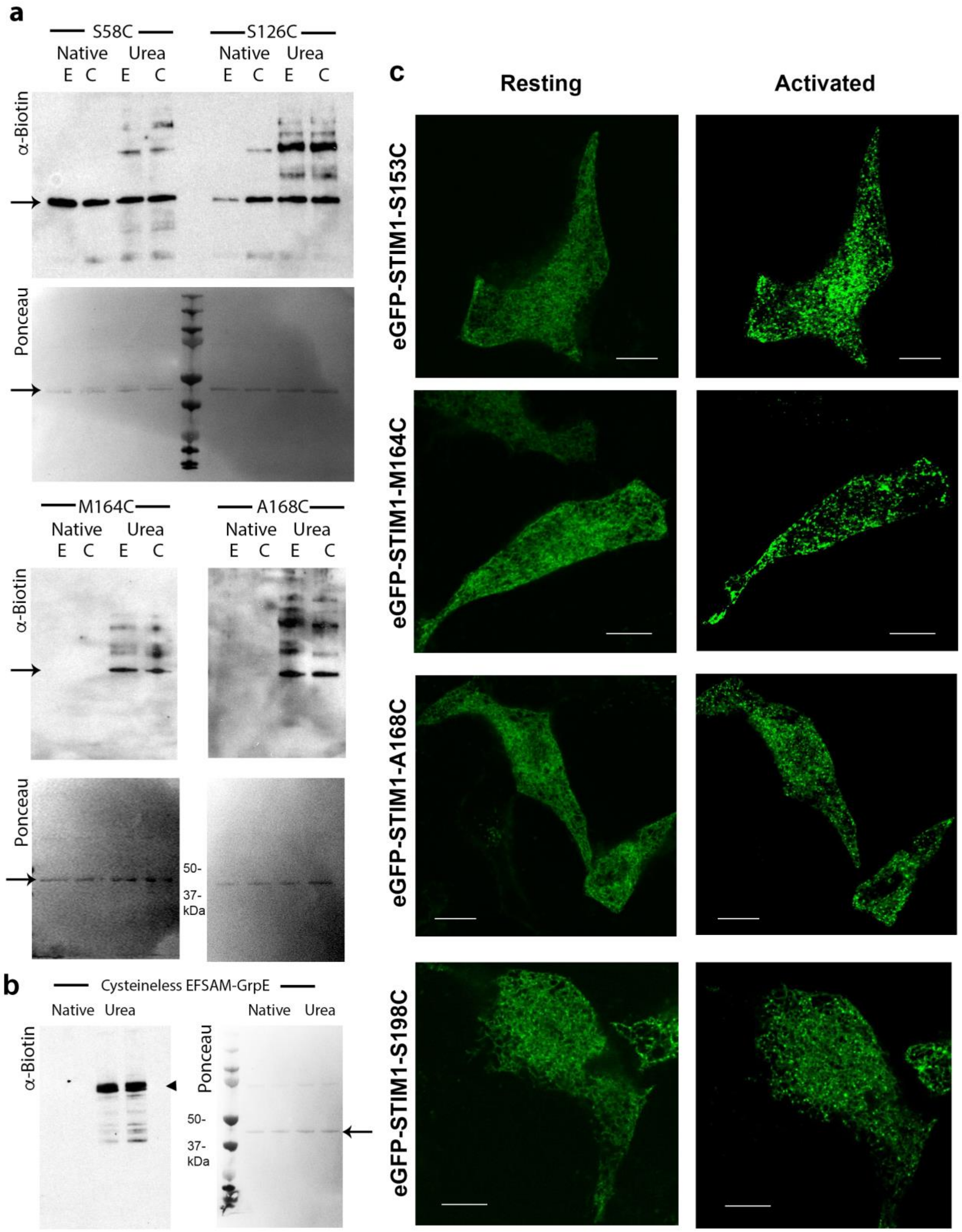

Supplementary Figure 11. Additional data from the EFSAM biotinylation experiments. a, Anti-biotin western blots of cysteine-substituted recombinant EFSAM-GrpE proteins (arrows), labelled by incubation with biotin-maleimide under native conditions or after denaturation with 6M urea, in the presence of EGTA (E) or Ca^{2+} (C). Ponceau staining shows equal protein loading across each set of samples, and arrows indicate the position of EFSAM-GrpE. The prominent upper band seen in urea-denatured samples is artifactual. b, Analysis of cysteineless EFSAM-GrpE labelled under the same conditions provides evidence that the upper band in urea-denatured samples (arrowhead) is an artifact. c, Confocal images of the representative cysteine-substituted GFP-STIM1 proteins STIM1(S153C), STIM1(M164C), STIM1(A168C), and STIM1(S198C) in resting cells and after store depletion. Scale bars, 5 μm.

Supplementary Fig. 12

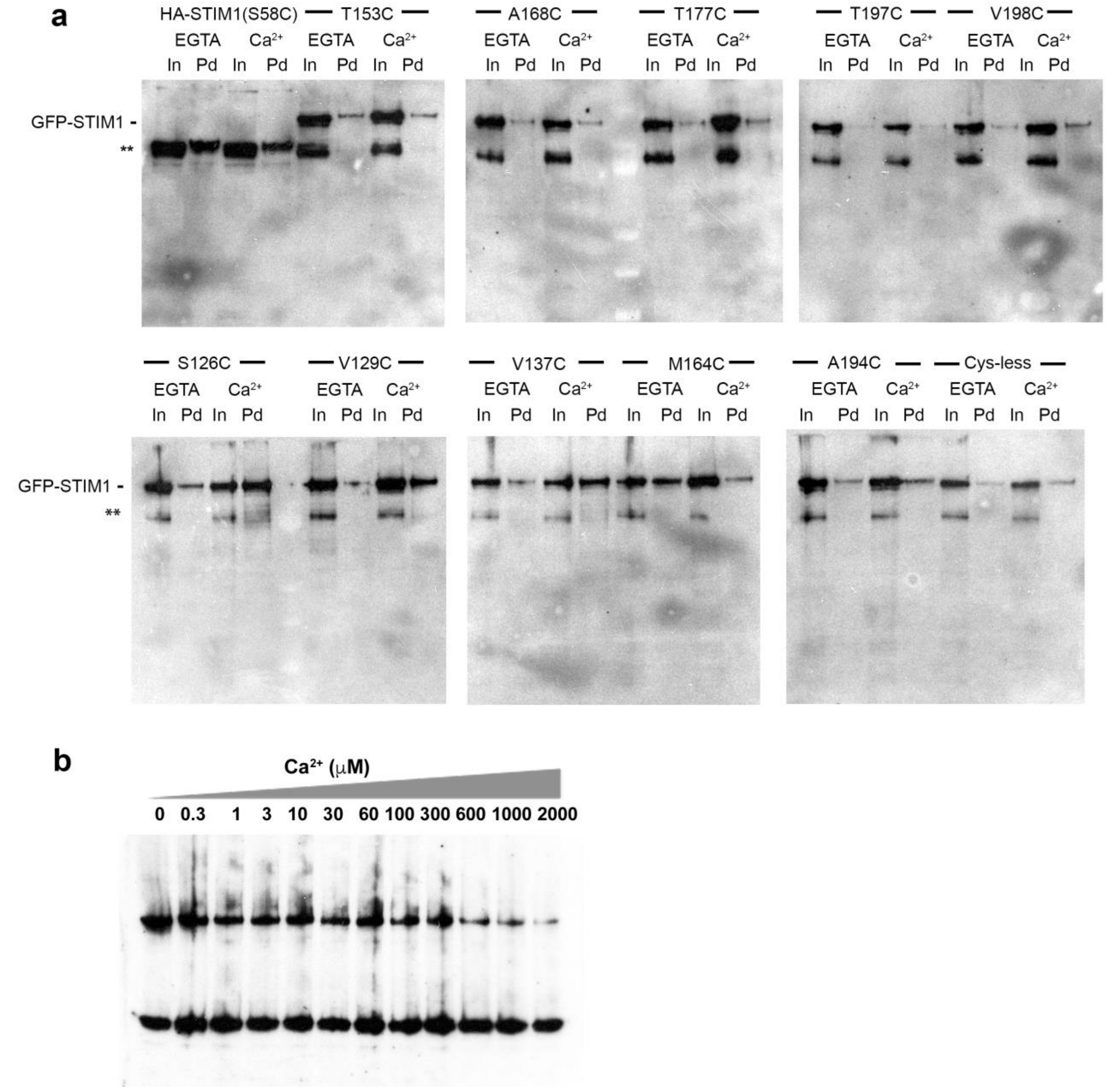

Supplementary Figure 12. Uncropped western blots corresponding to Figure 6b and Figure 7e.

Supplementary Table 1 Primers utilized for PCR amplification and mutagenesis.

Construct	Forward primer (5'-3')	Reverse primer (5'-3')
pET28-EFSAM-GrpE cloning:		
1. EFSAM (58-209)	GGAATTCCATATGAGTGAGGATGA GAAGCTCAGCTTTGA	GAGCTCGGATCCACTAGTAACGG CCGCCAG
2. GrpE amplicon	GAGCTCCATGCCATGGCTGAGGAT GAGAAG	AAGGAAAAAAGCGGCCGCTCACT TCTCCTCGCCCAC
pET28-EFSAM-GrpE D76A mutant	CATCCATAAGCTGATGGCTGACGA TGCCAATGGTG	
pET28- CGG-EFSAM-GrpE	AAGGAAAAAAGCGGCCGCTCACC GCTTCTCCTCGCCCACGGC	ACGCGTCGACATGGGATGCGGAG GCATGAGTGAG
pET28-EFSAM-SAH-GrpE (primers for linearizing the vector with BstB1 and AvrII sites	TACTGACCTAGGCTGGAGAAGGAC CTGGAGGCCGTGGGC	CAGTGCTTCGAAGAGCTCGGATC CACTAGTAACGGC
D76A-2NQ mutagenesis	AAACATCCATAAGCTGATGGCTAA CGATGCCAATGGTAATG	
Single-cysteine EFSAM-GrpE mutants:		
1. S58C	CGCGGCAGCCATATGTGTGAGGA TGAGAAGC	GCTTCTCATCCTCACACATATGGC TGCCGCG
2. S126C	GACCTGTGGAAGGCGTGGAAGTG CTCAGAAGTGTACAACTG	CAGTTGTACACTTCTGAGCACTT CCACGCCTTCCACAGGTC
3. T177C	ACACCACCATGACAGGGTGTGTAC TGAAGATGACAG	CTGTCATCTTCAGTACACACCCTG TCATGGTGGTGT
4. V129C	TGGAAGGCGTGGAAGTCATCAGAA TGCTACAACTGGACTGTGGATG	CATCCACAGTCCAGTTGTAGCATT CTGATGACTTCCACGCCTTCCA
5. V137C	TACAACTGGACTGTGGATGAGTGC ATACAGTGGCTCATTACGTAT	ATACGTAATGAGCCACTGTATGCA CTCATCCACAGTCCAGTTGTA
6. T153C	GCCACAGTATGAGGAATGCTTCCG GAAGTTGCAG	CTGCAACTTCCGGAAGCATTCCTC ATACTGTGGC
7. M164C	CTTACTGGCCACGCCTGCCCAAG GCTAGCAGTA	TACTGCTAGCCTTGGGCAGGCGT GGCCAGTAAG
8. A168C	GCCATGCCAAGGCTATGTGTAACC AACACCAC	GTGGTGTTGGTTACACATAGCCTT GGCATGGC
9. A194C	CAGAAGCTGCAGCTGAAGTGTCTG GACACAGTGCTGTT	AACAGCACTGTGTCCAGACACTTC AGCTGCAGCTTCTG
10. A197C	CAGCTGAAGGCCCTGGACTGCGT GCTGTTTGGGCCTC	GAGGCCCAAACAGCACGCAGTCC AGGGCCTTCAGCTG
11. V198C	CTGAAGGCCCTGGACACATGCCT GTTTGGGCCTCCTCTC	GAGAGGAGGCCCAAACAGGCATG TGTCCAGGGCCTTCAG
HA-eGFP-STIM1 with subcloned NQ mutants:		
1. NQ amplicon	CATGGCCGTACGAAACATCCATAA GCTGATGGAT	TCAGACGCGTCAAGAGAGGAGGC CCAAACAGCACTGTGTC
2. Vector amplicon	TCAGACGCGTCATAATCACCTCAA GGACTTCATGCTGGTG	CATGACGTACGGCCTCGAAGCTG AGTTTCTCATCCTCACTGTG

Single-cysteine eGFP-STIM1 mutants:		
1. S126C	GACCTGTGGAAGGCATGGAAGTGCTCAGAAGTATACAATTG	CAATTGTATACTTCTGAGCACTTCCATGCCTTCCACAGGTC
2. T177C	ACACCACCATGACAGGGTGTGTGCTGAAGATGACAG	CTGTCATCTTCAGCACACACCCTGTCATGGTGGTGT
3. V129C	TGGAAGGCATGGAAGTCATCAGAATGCTACAATTGGACCGTGGATG	CATCCACGGTCCAATTGTAGCATTCTGATGACTTCCATGCCTTCCA
4. V137C	TACAATTGGACCGTGGATGAGTGCGTACAGTGGCTGATCACATAT	ATATGTGATCAGCCACTGTACGCACTCATCCACGGTCCAATTGTA
5. T153C	GCCTCAGTATGAGGAGTGCTTCCGGAAGCTGCAG	CTGCAGCTTCCGGAAGCACTCCTCATACTGAGGC
6. M164C	CTCAGTGGCCATGCCTGCCCAAGGCTGGCTGTC	GACAGCCAGCCTTGGGCAGGCATGGCCACTGAG
7. A168C	GCCATGCCAAGGCTGTGTGTCACCAACACCAC	GTGGTGTTGGTGACACACAGCCTTGGCATGGC
8. A194C	CAGAAGCTGCAGCTGAAGTGTCTGGATACAGTGCTCTT	AAGAGCACTGTATCCAGACACTTCAGCTGCAGCTTCTG
9. A197C	CAGCTGAAGGCTCTGGATTGCGTGCTCTTTGGGCCTC	GAGGCCCAAAGAGCACGCAATCCAGAGCCTTCAGCTG
10. V198C	CTGAAGGCTCTGGATACATGCCTCTTTGGGCCTCCTCTC	GAGAGGAGGCCCAAAGAGGCATGTATCCAGAGCCTTCAG
pET28-STIM2-EFSAM-GrpE	GGGAATTCCATATGACTGAAGAAGACAGGTTTAGCTTGGAA	ATCGGGAATTCGCCCTTGACAGCTCGAGGTTATGAGGTGGGCGTGTCAGAGG
pEGFP-N1-STIM2(1-434)-WT-GFP	CCGCTCGAGATG AACGCAGCCGGGATCAGA	CGG GGATCCCCAGACCAACTGCTTCTCAGTTCAAA
pEGFP-N1-STIM2(1-434)-D167A-GFP mutant	CAAACAATACATAAACAAATGGCTGATGACAAAGATGGTGGAATTG	
pET28-EFSAM-GrpE (6-11):		
1	AAGTGATGAGTTCCTAAGGCAGGACCTCAATTACCATAACC	
2	CATAGCACCTTCCATGGTSAGRATAAGCTTATCAGCGTG	
3	TAAGCTTATCAGCGTGSAGRACCTGTGGAAGGCGTG	
pEX-SP-CFP/YFP-EFSAM-TM-CC1-GrpE:		
1. CFP/YFP STIM1-TM-CC1 amplicon	CGGGGTACC ATGGATGTATGCGTCCGTCTTGCC	CTGACCGGT AGCATACCATGAGCTGTGAGATTCCAGCTC
2. GrpE amplicon	CTGACCGGTCTGGAGAAGGACCTGGAGGCCGTGGGCCAG	AAGGAAAAAAGCGGCCGCC TCACCGCTTCTCCTCGCCCACGGCCACCCG

Supplementary Note 1

More detailed characterization of STIM1-2NQ

STIM1-2NQ, expressed at moderate levels, had an ER distribution in most resting cells (Fig. 4b). In a minor fraction of resting cells, STIM1-2NQ fluorescence was partially localized to small puncta. These few small puncta need not be given undue weight, since a fraction of wildtype STIM1 may localize to puncta in resting cells, especially when the recombinant protein is expressed at high levels. In sharp contrast to the wildtype protein, though, and regardless of expression level, STIM1-2NQ showed very little relocalization to ER-plasma membrane junctions after store depletion (Fig. 4b). Notably, whereas STIM1(D76A) localizes in puncta constitutively[4], incorporation of the D76A mutation into STIM1-2NQ did not override its failure to form puncta (Supplementary Fig. 4). This finding ruled out the possibility that STIM1-2NQ binds Ca^{2+} more tightly at the EF-hand site than wildtype STIM1 and shifts the threshold for activation to lower ER-luminal Ca^{2+} concentrations that were not reached under our experimental conditions. STIM1-2NQ is simply very poorly responsive in cells.

EFSAM-2NQ far-UV CD spectra documented an □-helix content comparable to wildtype EFSAM-GrpE and a secondary structure unaffected by the presence or absence of Ca^{2+} (Fig. 5b), yet ITC showed that only a single Ca^{2+}-binding site was occupied in the concentration range monitored (Fig. 4c). A straightforward explanation of the findings would be that negatively charged sidechains in region 2 are directly involved in coordinating Ca^{2+}. An alternative explanation, keeping in mind the allosteric effect of the STIM1(D76A) replacement on Ca^{2+} binding at other sites, would be that the negatively charged sidechains in region 2 are required for Ca^{2+} binding elsewhere in EFSAM.

Supplementary Note 2

STIM1-2NQ and STIM2-2NQ maintain an inactive conformation despite Ca^{2+} depletion

The simplest mechanism that would explain the failure to relocalize is that STIM1-2NQ is unable to transition to the active conformation upon loss of Ca^{2+} from the EF-hand sites. We examined this question directly in ER membranes, using an oxidative crosslinking assay that measures apposition of the STIM1 transmembrane helices[5]. STIM1(A230C)-2NQ showed minimal crosslinking in this assay compared to 'wildtype' control STIM1(A230C), indicating that it maintained the inactive conformation even in the absence of Ca^{2+} (Supplementary Fig. 5a,b). Introducing the D76A mutation into STIM1-2NQ did not override the failure to crosslink (Supplementary Fig. 5c), which established that the impairment was not a failure of Ca^{2+} dissociation from the EF-hand, in line with the localization result for GFP-STIM1(D76A)-2NQ (Supplementary Fig. 4).

A second direct indicator of the STIM active conformation is the release of SOAR/CAD from its intramolecular interaction with CC1. The clearest demonstration of this mechanism is that the ER-tethered recombinant fragment EFSAM-TM-CC1 retains soluble SOAR/CAD near the ER in resting cells, and releases SOAR/CAD upon store depletion[6,7]. A modification of this assay takes advantage of the fact that a fraction of EFSAM-TM-CC1 expresses at the cell surface, with the luminal EFSAM domain exposed on the extracellular side, allowing precise experimental control over the Ca^{2+}

concentration ‘seen’ by the luminal domain and the resulting conformational change (Supplementary Fig. 6). We used STIM2 EFSAM-TM-CC1 for this experiment, because the STIM2 EFSAM-TM-CC1 fragment was more efficiently expressed at the cell surface than the corresponding fragment of STIM1. The Ca^{2+}-dependent association of SOAR/CAD with STIM2 EFSAM-TM-CC1 is illustrated in Supplementary Figs. 6c and 6d. Note that the midpoint Ca^{2+} concentration is ~2 mM, contrasting with ~400 μM for STIM2 in the ER of intact cells[8] and affording further evidence that Ca^{2+} binding to EFSAM is influenced by the context. ‘Context’ in this case could encompass variables including the entropic cost of capturing the independent SOAR/CAD fragment, differences in lipid composition or thickness between ER membrane and plasma membrane, and the presence or absence of specific protein partners.

In initial experiments, we verified by ITC that STIM2 EFSAM, like STIM1 EFSAM, has multiple Ca^{2+}-binding sites (Supplementary Fig. 7a). We then introduced D>N and E>Q substitutions into STIM2 EFSAM-TM-CC1-YFP at positions corresponding to the sites mutated in STIM1-2NQ, expressed the protein in cells, and examined the recruitment of mCherry-SOAR/CAD to the plasma membrane by EFSAM-TM-CC1 under low-Ca^{2+} and high-Ca^{2+} conditions. Wildtype STIM2 EFSAM-TM-CC1-YFP and its D167A EF-hand mutant (corresponding to D76A in STIM1) served as controls. The results were clearcut (Supplementary Fig. 7b–g). Wildtype STIM2 EFSAM-TM-CC1 recruited SOAR/CAD poorly in the absence of extracellular Ca^{2+}, but bound SOAR/CAD efficiently when the external Ca^{2+} concentration was raised to 12 mM. The EF-hand mutant STIM2(D167A) failed to recruit appreciable SOAR/CAD regardless of Ca^{2+} concentration. And STIM2-2NQ retained SOAR/CAD at the plasma membrane at both low and high Ca^{2+} concentrations.

We conclude that STIM1-2NQ and STIM2-2NQ have minimal ability to assume an active conformation, even in the absence of Ca^{2+}. Mechanistically, the 2NQ mutations might interfere directly or indirectly with the known EFSAM–EFSAM dimer interaction underlying activation, or might impair a still unidentified interaction, for example at an EFSAM–lipid interface.

Supplementary Note 3

Further evidence of a rearrangement in the region ^{121}WKAWK—QWLIT^{143}

There were two noteworthy exceptions to the generalizations that H-D exchange of EFSAM(D76A) was insensitive to Ca^{2+}, and that exchange of EFSAM-2NQ matched exchange of wildtype EFSAM in the presence of Ca^{2+}. First, H-D exchange into the ^{136}EVIQWLIT^{143} peptide of EFSAM(D76A) was affected, albeit very mildly, by the presence of Ca^{2+} (Fig. 5f, Supplementary Fig. 9b). Second, exchange in the region ^{121}WKAWK—QWLIT^{143} in EFSAM-2NQ deviated from that measured in wildtype EFSAM (Fig. 5h, Supplementary Fig. 9c). These exceptions reinforce the HDX–MS findings for wildtype EFSAM that indicate the region of protein backbone encompassing residues 121-143 is particularly susceptible to dynamic rearrangement.

Supplementary Note 4

Intrinsic and extrinsic fluorescent probes of EFSAM structure

Coincidentally, the four tryptophan residues of EFSAM are in the region marked by most prominent changes in the HDX–MS signals (Fig. 5j, Supplementary Fig. 9d). There are no tryptophan residues in GrpE, so we were able to monitor the intrinsic tryptophan fluorescence of EFSAM for additional confirmation that there is a Ca^{2+}-dependent conformational change in this region. Indeed, wildtype EFSAM displayed an enhancement of tryptophan fluorescence compared to the denatured protein both in the absence and in the presence of Ca^{2+}, indicating that the tryptophan sidechains are at least partly buried in both conditions. Further, an increase in fluorescence confirmed that the local environment of one or more tryptophan residues in EFSAM did change on binding of Ca^{2+} (Supplementary Fig. 10a–c). The slight Ca^{2+}-dependent alteration in peptide backbone dynamics of this region in EFSAM(D76A) was not detected as a tryptophan fluorescence change.

ANS (8-anilinonaphthalene-1-sulfonic acid) is a commonly used extrinsic probe of protein conformational change. Wildtype EFSAM-GrpE, EFSAM(D76A)-GrpE, and EFSAM-2NQ-GrpE all bound ANS as judged by the enhanced fluorescence and a blue shift in the fluorescence emission peak of ANS–protein samples compared to free ANS (Supplementary Fig. 10d–f). The three protein variants showed distinct patterns of change in response to titration with Ca^{2+} in the range 1 μM to 1 mM, discussed here in order of increasing complexity. EFSAM(D76A) showed no change in ANS fluorescence when Ca^{2+} was added. EFSAM-2NQ exhibited modest quenching of ANS fluorescence. Wildtype EFSAM exhibited both modest quenching, evident at somewhat lower Ca^{2+} concentrations than for EFSAM-2NQ, and a further 12-nm blue shift of fluorescence emission in the presence of higher concentrations of Ca^{2+}. The latter change in the emission spectrum indicates decreased polarity surrounding the ANS binding site(s) or availability of new nonpolar site(s). The blue shift is completed at ~300 μM Ca^{2+}, reminiscent of results from the D4 binding competition assay, and reinforcing the conclusion that Ca^{2+} binding to EFSAM occurs in this concentration range.

These experiments with intrinsic and extrinsic fluorescence probes support the conclusion from CD and HDX–MS data that there are structured conformations of EFSAM at both endpoints of the high Ca^{2+}–low Ca^{2+} conformational change observed by FRET in Fig. 1g. The conformational change monitored by EFSAM–EFSAM FRET is substantially completed between 0 μM Ca^{2+} and 30 μM Ca^{2+}, the concentration used in the HDX–MS experiments. The D4 Ca^{2+} sensor titration experiments (Fig. 3g,i) and ANS fluorescence measurements (Supplementary Fig. 10d) indicate that further Ca^{2+} binding to wildtype EFSAM occurs at Ca^{2+} concentrations up to 300–400 μM, and this may correspond to a further consolidation of the inactive conformation.

SUPPLEMENTARY REFERENCES

1 Nakamura, A., Takumi, K., and Miki, K. Crystal structure of a thermophilic GrpE protein: insight into thermosensing function for the DnaK chaperone system. *J. Mol. Biol.* 396, 1000–1011 (2010).

2 Gelinas, A.D., Langsetmo, K., Toth, J., Bethoney, K.A., Stafford, W.F., and Harrison, C.J. A structure-based interpretation of E. coli GrpE thermodynamic properties. *J. Mol. Biol.* 323, 131–142 (2002).

3 Gelinas, A.D., Toth, J., Bethoney, K.A., Langsetmo, K., Stafford, W.F., and Harrison, C.J. Thermodynamic linkage in the GrpE nucleotide exchange factor, a molecular thermosensor. *Biochemistry* 42, 9050–9059 (2003).

4 Liou, J., Kim, M.L., Heo, W.D., Jones, J.T., Myers, J.W., Ferrell, J.E., Jr., and Meyer, T. STIM is a Ca^{2+} sensor essential for Ca^{2+}-store-depletion-triggered Ca^{2+} influx. *Curr. Biol.* 15, 1235–1241 (2005).

5 Hirve, N., Rajanikanth, V., Hogan, PG, and Gudlur, A. Coiled-coil formation conveys a STIM1 signal from ER lumen to cytoplasm. *Cell Rep.* 22, 72–83 (2018).

6 Ma, G., Wei, M., He, L., Liu, C., Wu, B., Zhang, S.L., Jing, J., Liang, X., Senes, A., Tan, P., Li, S., Sun, A., Bi, Y., Zhong, L., Si, H., Shen, Y., Li, M., Lee, M.S., Zhou, W., Wang, J., Wang, Y., and Zhou, Y. Inside-out Ca^{2+} signalling prompted by STIM1 conformational switch. *Nat. Commun.* 6:7826 (2015).

7 Korzeniowski, M.K., Wisniewski, E., Baird, B., Holowka, D.A., and Balla, T. Molecular anatomy of the early events in STIM1 activation oligomerization or conformational change. *J. Cell Sci.* 130, 2821–2832 (2017).

8 Brandman, O., Liou, J., Park, W. S., and Meyer, T. STIM2 is a feedback regulator that stabilizes basal cytosolic and endoplasmic reticulum Ca^{2+} levels. *Cell* 131, 1327–1339 (2007).
